# Response to the comment on: “Diabetic retinopathy and diabetic kidney disease, either isolated or associated, impact on the 10-year risk of cardiovascular disease: are we dealing with similar conditions?”

**DOI:** 10.20945/2359-4292-2025-0390

**Published:** 2025-10-08

**Authors:** Clara Krummenauer Maraschin, Janine Alessi, Mateus Augusto dos Reis, Gabriela Oliveira Gonçalves Molino, Gabriela Heiden Teló, Beatriz D. Schaan

**Affiliations:** 1 Universidade Federal do Rio Grande do Sul, Porto Alegre, RS, Brasil; 2 Faculdade de Medicina, Pontifícia Universidade Católica do Rio Grande do Sul, Porto Alegre, RS, Brasil; 3 Universidade Feevale, Novo Hamburgo, RS, Brasil; 4 Universidade Federal de Ciências da Saúde de Porto Alegre, Porto Alegre, RS, Brasil; 5 Instituto de Avaliação de Tecnologia em Saúde (IATS) - Conselho Nacional de Desenvolvimento Científico e Tecnológico (CNPq), Brasil; 6 Serviço de Endocrinologia, Hospital de Clínicas de Porto Alegre, Porto Alegre, RS, Brasil

Dear Editor,

We thank the colleague for their thoughtful comments and the opportunity to respond.

Regarding the analysis of participants with type 1 diabetes, we would like to highlight that this limitation was acknowledged in the discussion section of the manuscript, where we stated: “the lack of associations between the complications of type 1 diabetes and the 10-year risk of ASCVD may be attributed to the study’s limited power, owing to a small sample size, which makes this analysis exploratory” ^(1)^.

We respectfully disagree, however, with the assertion that only two variables were considered in the risk prediction, and that important factors such as lipid control were not evaluated. On the contrary, lipid profile measurements were transparently presented in Table 1 and included in the outcome analysis, as they are components of the risk calculation. As clarified in the manuscript, the 10-year atherosclerotic cardiovascular disease (ASCVD) risk was calculated using the American College of Cardiology’s risk estimator, which incorporates the following variables: age, sex, race, total cholesterol, HDL cholesterol, systolic blood pressure, treatment for hypertension, diabetes status, and smoking status.

We once again thank the colleague for their engagement and the opportunity to clarify these points.

Sincerely,

Beatriz D. Schaan, on behalf of the authors

## Data Availability

datasets related to this article will be available upon request to the corresponding author.
